# Supplementary material for: Transcriptional Targeting of Dendritic Cells Using an Optimized Human Fascin1 Gene Promoter
Source: Int J Mol Sci. 2023 Nov 29;24(23):16938. doi: 10.3390/ijms242316938 (PMC10706967; doi:10.3390/ijms242316938)
Supplement: Supplementary file 1 [file ijms-24-16938-s001.zip › ijms-2713775-supplementary.pdf]

Transcriptional Targeting of Dendritic Cells Using an Optimized Human *Fascin1* Gene Promoter

Yanira Zeyn[1], Dominika Hobernik[1], Ulrich Wilk[2], Jana Pöhmerer[2], Christoph Hieber[1], Carolina Medina-Montano[1], Nadine Röhrig[1], Caroline F. Strähle[3], Andrea K. Thoma-Kress[3], Ernst Wagner[2], Matthias Bros[1,*,+], and Simone Berger[2,*,+]

[1] Department of Dermatology, University Medical Center of the Johannes Gutenberg University (JGU) Mainz, 55131 Mainz, Germany
[2] Pharmaceutical Biotechnology, Department of Pharmacy, Center for NanoScience, Ludwig-Maximilians-Universität (LMU) Munich, 81377 Munich, Germany
[3] Institute of Clinical and Molecular Virology, Friedrich-Alexander-Universität (FAU) Erlangen-Nürnberg, 91054 Erlangen, Germany

* Correspondence: mbros@uni-mainz.de (M.B.); simone.berger@cup.uni-muenchen.de (S.B.)

\+ These authors contributed equally to this work.

Supplementary materials

Supporting figures **2–7**

Supporting tables **8-10**

Supporting figures

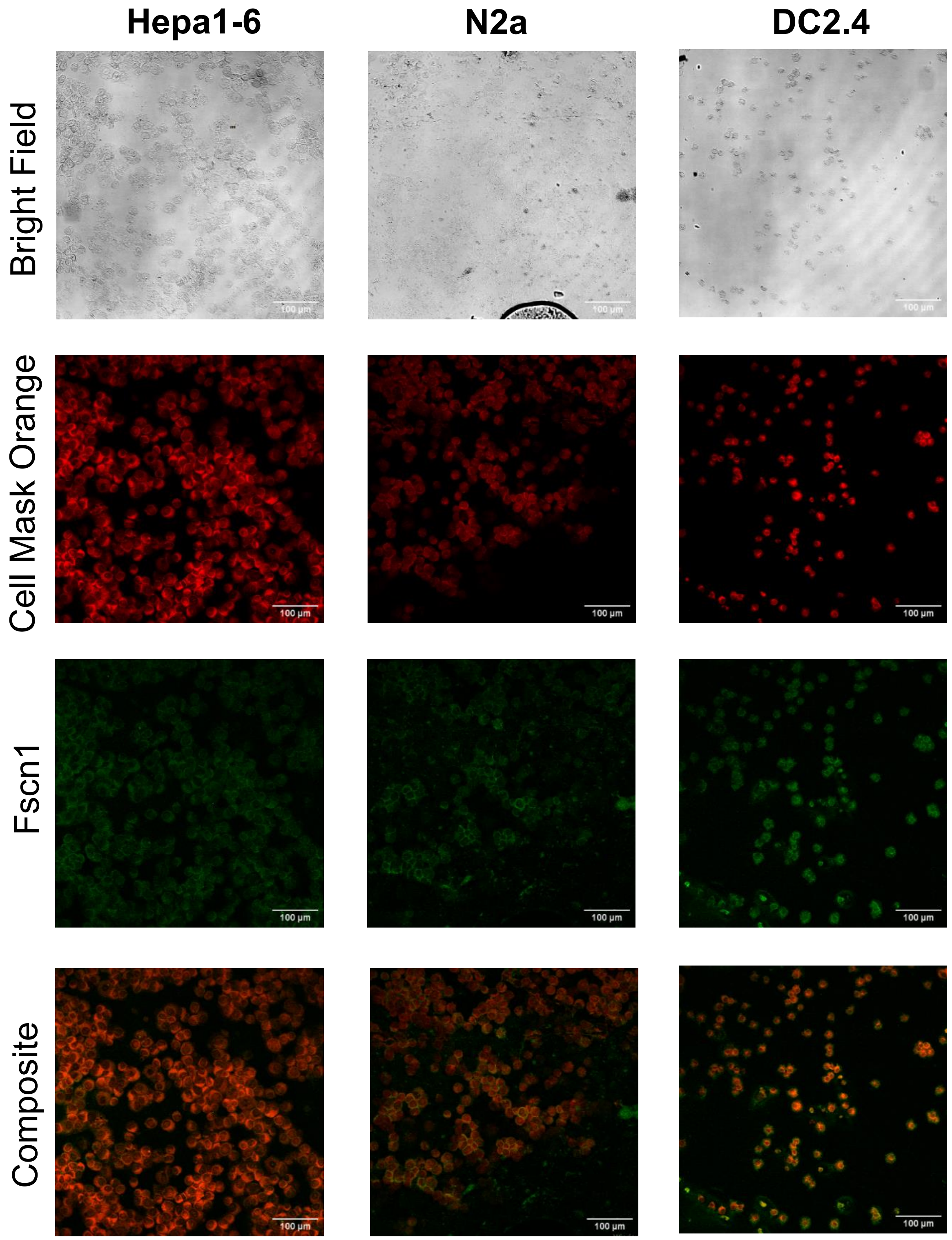

Figure S1. *Fascin1* (*Fscn1*) expression in DC2.4 and various tumor cell lines (all of murine origin). CLSM images of cytospins of Hepa1-6 (*left column*), N2a (*middle column)* and DC2.4 cells (*right column*) show cells in bright field (*top* row), cell margins by using Cell Mask Orange (*second row*), *Fscn1* expression detected using antibodies (*third row*) and a composite of both (*lower row*). Intensity of Fscn1 staining was adapted to isotype control (*data not shown*). Scale bar, 100 µm.

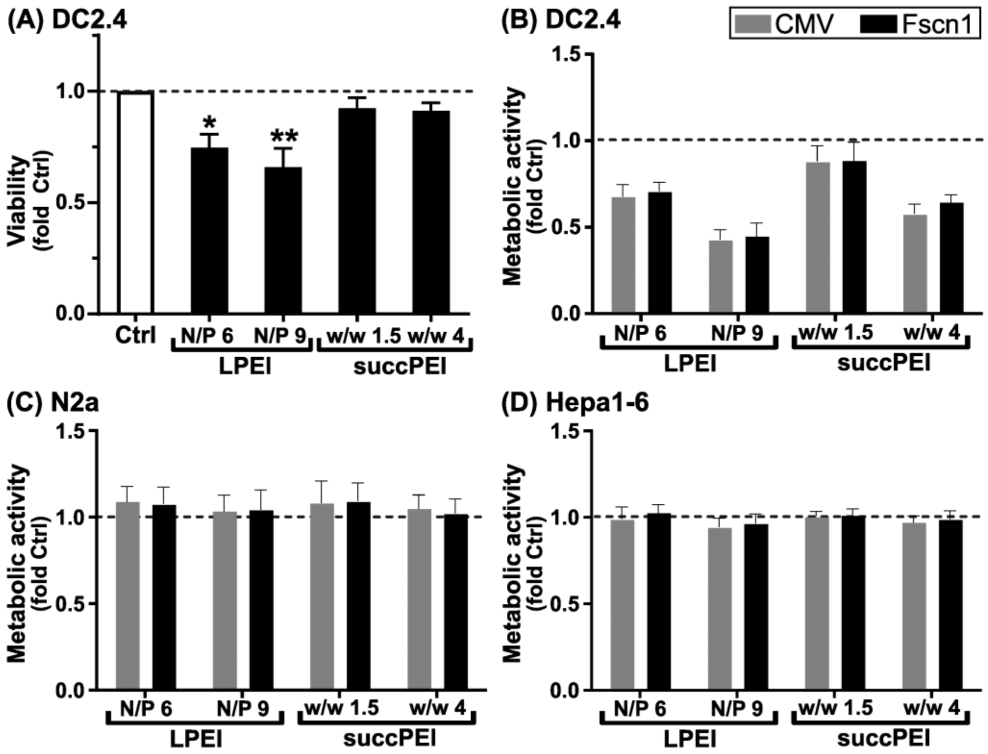

Figure S2. Cellular effects of transfection of *Fscn1*-expressing murine cell types with polyplexes. (**A**) Viability and (**B**-**D**) metabolic activity of *Fscn1*-expressing cell lines in relation to HBG-buffer treated control cells at 24 h after incubation with polyplexes. DC2.4 cells were incubated with 1 µg pFscnLuc per 100,000 cells (**A**), and 500 ng of either plasmid (pCMV-Luc, *grey*; pFscnLuc, *black*) per 5000 cells (**B**); the neuroblastoma cell line N2a (**C**) and the hepatoma cell line Hepa1-6 (**D**) both at a cell density of 10,000 cells were treated with a pDNA concentration of 200 ng/well. In all cases, pDNA was complexed in parallel with LPEI (N/P 6 and 9, resp.) and succPEI (w/w 1.5 and 4, resp.). (**A**) After overnight incubation, samples were harvested, incubated with eFl780-FVD (fixable viability dye) to delineate dead cells and subjected to flow cytometric analysis. The graph denotes the frequencies of FVD-negative viable cells (n=4; mean + SEM), normalized to untreated cells (Ctrl). Significant differences versus Ctrl: * $p \leq 0.05$; ** $p \leq 0.01$ (one-way ANOVA, Tukey test; GraphPad Prism™ 9.5.1). (**B-D**) Data denote the relative metabolic activity (n=3; mean + SD) given as fold of the control (Ctrl; HBG buffer treated cells).

Note: For succPEI, w/w ratios of 1.5 and 4 represent N/P ratios of ~ 11.25 and ~ 30 of an unsubstituted PEI.

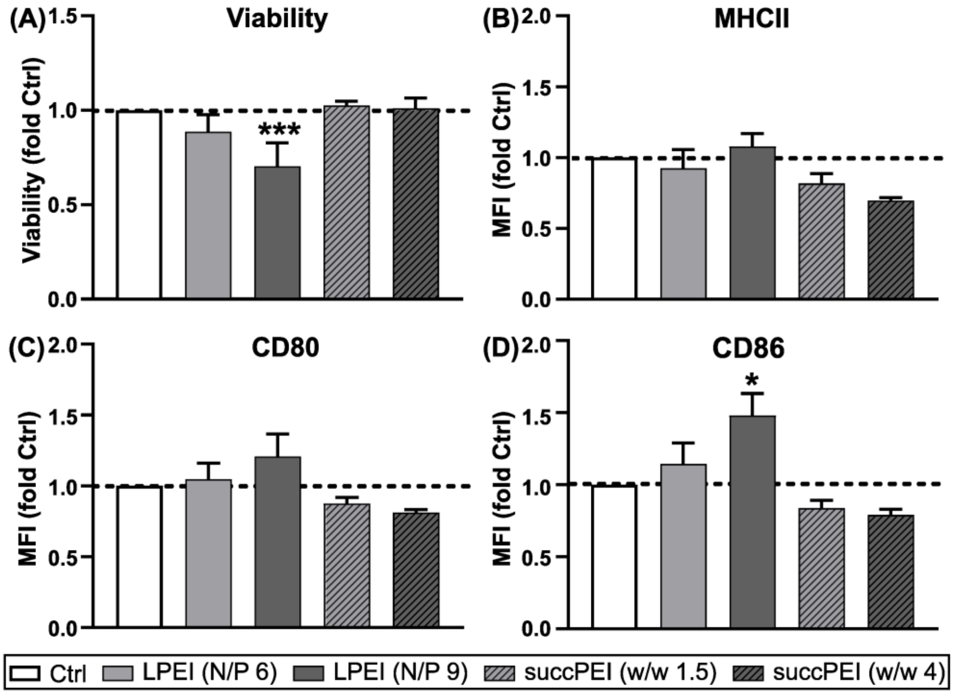

Figure S3. Viability and activation marker expression of BMDC after incubation with LPEI and succPEI polyplexes. BMDCs (20,000 cells per well) were incubated overnight with LPEI (N/P 6 and 9, resp.) and succPEI (w/w 1.5 and 4, resp.) polyplexes at a concentration of 1 μg pFscnLuc/well. On the next day, samples were harvested, and both viability and activation marker surface expression were assayed by flow cytometry. Graph **A** denotes the frequencies of FVD (fixable viability dye)-negative viable cells (n=4; mean + SEM), normalized to untreated cells (Ctrl). Graphs **B-D** denote the mean fluorescence intensity (MFI) (n=4; mean + SEM) in relation to the control (untreated cells). Significant differences versus Ctrl: * $p \leq 0.05$; *** $p \leq 0.001$ (one-way ANOVA, Tukey test; GraphPad Prism™ 9.5.1). MHCII, major histocompatibility complex class 2; CD80 and CD86, costimulatory receptors for T cell activation.

Note: For succPEI, w/w ratios of 1.5 and 4 represent N/P ratios of ~ 11.25 and ~ 30 of an unsubstituted PEI.

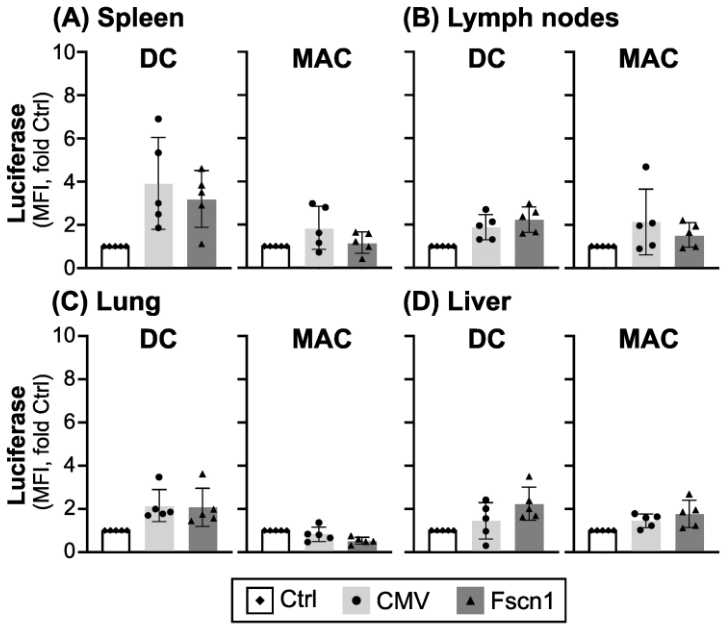

Figure S4. *In vivo* comparison of *Fscn1* and CMV promoter activities on single cell level in BALB/c mice. *Ex vivo* analysis of single cell suspensions retrieved from different organs at 24 h after intravenous injection of 200 µL of succPEI polyplexes (w/w 1.5; HBG), containing 60 µg pDNA. Spleen (**A**), lymph nodes (**B**), lung (**C**), and liver non-parenchymal cells (NPC, **D**) were processed to single cell suspensions, stained, and subjected to flow cytometric analyses to monitor the luciferase signal. The gating strategy for (**A–C**) is described in Figure S5. The gating strategy for (**D**) is described in Figure S6. The mean fluorescence intensity (MFI) of luciferase expression is displayed in relation to HBG buffer treated animals (Ctrl) (n=5, mean ± SEM). DC, dendritic cells; MAC, macrophages.

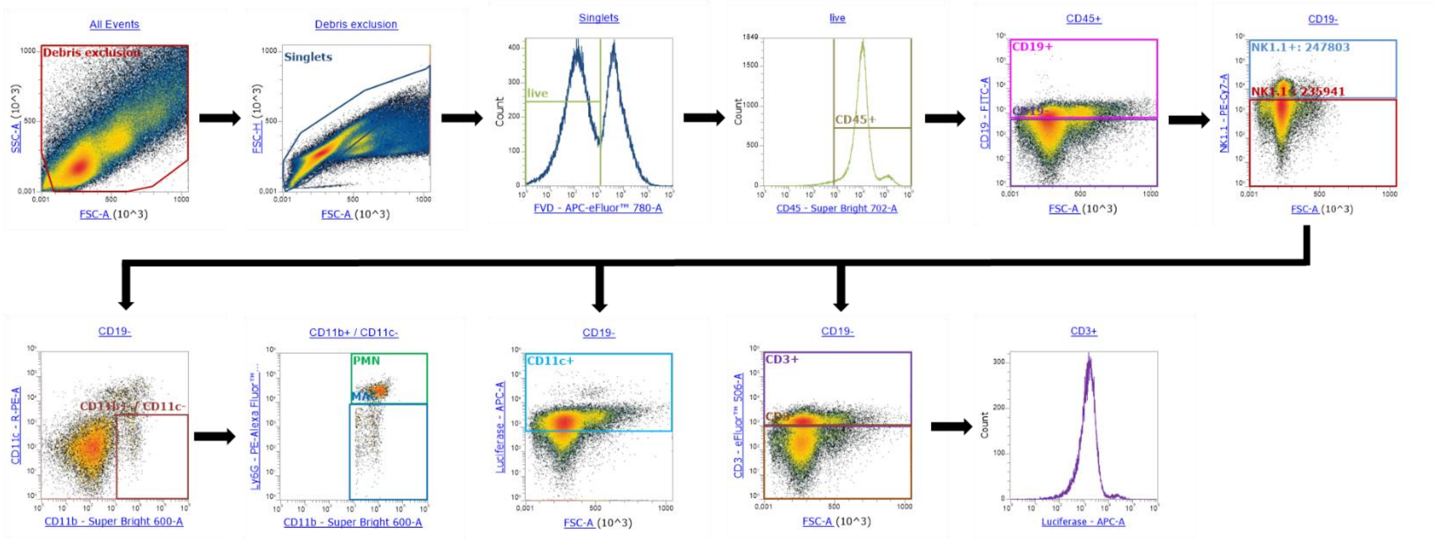

Figure S5. Gating strategy for immune cells in spleen, lymph nodes, and lungs. First, debris and doublets were excluded from further analysis. Then, cells were sequentially gated for FVD^{-} and CD45^{+} fractions, to include only living immune cells. Next, B cells were identified as CD19^{+}. The CD19^{-} fraction was further discriminated in NK1.1^{+} (NK cells) and NK1.1^{-} fraction. Of the NK1.1^{-} fraction, cell populations were identified as follows: CD11c^{+} (DC, dendritic cells), CD3^{+} (T cells), CD11c^{-}/CD11b^{+}/Ly6G^{+} (PMN, polymorphonuclear neutrophils), and CD11c^{-} /CD11b^{+}/Ly6G^{-} (MAC, macrophages). For all identified immune cell populations the mean fluorescence intensity (MFI) of luciferase expression detected via intracellular staining with luciferase-specific antibody was determined.

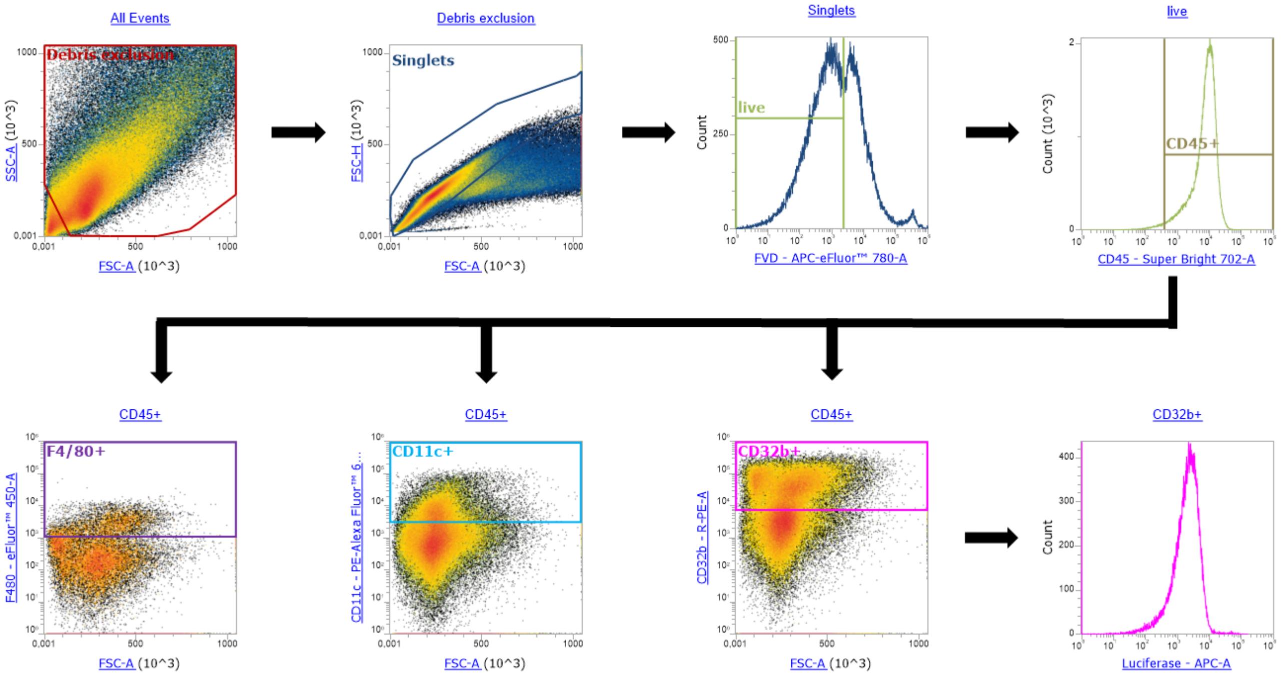

Figure S6. Gating strategy of liver immune cells. First, debris and doublets were excluded from further analysis. Then, cells were sequentially gated for FVD⁻ and CD45⁺ fractions, to include only living immune cells. Macrophages (KC; Kupffer cells) were identified as F4/80⁺, DCs as CD11c⁺, and liver sinusoidal endothelial cells (LSEC) as CD32b⁺. For all identified immune cell populations the mean fluorescence intensity (MFI) of luciferase expression detected via intracellular staining with luciferase-specific antibody was determined.

Supporting tables

Table S1. DLS and ELS measurements of LPEI and succPEI pDNA polyplexes.

		**Z-average [nm]**		**Number mean [nm]**		**PdI**		**Zeta-potential [mV]**	
		mean	SD	mean	SD	mean	SD	mean	SD
Different pDNA types (10 µg/mL pDNA)									
**pCMVLuc**	**LPEI (N/P 6)**	61.6	3.2	40.5	6.4	0.300	0.039	13.1	1.9
	**SuccPEI (w/w 1.5)**	54.1	2.0	35.0	3.4	0.248	0.027	11.4	1.7
**pFscnLuc**	**LPEI (N/P 6)**	70.6	0.6	47.4	2.3	0.138	0.012	11.4	1.4
	**SuccPEI (w/w 1.5)**	63.6	0.4	40.8	2.2	0.191	0.004	8.7	0.6
Different pDNA concentrations (*in vitro*, *in vivo*)									
**10** µg/mL pCMVLuc	**LPEI (N/P 6)**	88.1	2.9	38.8	6.0	0.286	0.036	11.3	2.0
	**LPEI (N/P 9)**	105.4	1.0	47.9	15.1	0.253	0.010	20.4	3.1
	**SuccPEI (w/w 1.5)**	98.0	4.1	48.9	6.9	0.233	0.015	11.4	0.4
	**SuccPEI (w/w 4)**	77.5	3.0	32.8	2.1	0.265	0.007	23.2	2.3
**25** µg/mL pCMVLuc	**LPEI (N/P 6)**	84.0	0.5	37.6	9.6	0.213	0.017	22.3	1.5
	**LPEI (N/P 9)**	72.4	0.1	44.4	2.8	0.159	0.014	17.5	2.7
	**SuccPEI (w/w 1.5)**	97.5	1.0	43.4	17.2	0.213	0.007	16.8	0.8
	**SuccPEI (w/w 4)**	68.7	0.6	38.7	0.6	0.172	0.015	15.5	1.8
**300** µg/mL pCMVLuc	**LPEI (N/P 9)**	93.5	0.8	52.4	1.7	0.180	0.024	29.1	1.6
	**succPEI (w/w 1.5)**	138.3	5.3	89.4	3.5	0.147	0.009	25.7	1.4

DLS and ELS measurements (n=3, mean ± SD) of pDNA polyplexes formed with different pDNA types at indicated N/P (LPEI) or w/w (succPEI) ratios, respectively, and at different pDNA concentrations of 10 µg mL^{-1} (referring to *in vitro* transfections in tumor cell lines N2a and Hepa1-6), 25 µg mL^{-1} (referring to *in vitro* transfections in DC2.4 cells), and 300 µg mL^{-1} (referring to *in vivo* experiments).
Note: For succPEI, w/w ratios of 1.5 and 4 represent N/P ratios of ~ 11.25 and ~ 30 of an unsubstituted PEI.

Table S2. Significance levels and fold-changes of the data displayed in Fig. 4A.

		**Significance levels**		**Fold-change of RLU**	
		p value			
**LPEI**	Lung	0.0377	*	10.4	(-)
	Liver	0.0174	*	10.8	(-)
CMV vs *Fscn1*	Spleen	0.8585	ns	1.1	(+)
**succPEI**	Lung	0.8632	ns	1.1	(-)
	Liver	0.6982	ns	1.2	(-)
CMV vs *Fscn1*	Spleen	0.0102	*	12.6	(+)
**CMV**	Lung	0.0284	*	210.0	(-)
	Liver	0.0166	*	11.5	(-)
LPEI vs succPEI	Spleen	0.0584	ns	8.1	(-)
**Fscn1**	Lung	0.0500	*	21.8	(-)
	Liver	0.4268	ns	1.3	(-)
LPEI vs succPEI	Spleen	0.2708	ns	1.4	(+)

Luciferase expression in organs (*n*=5; mean + SD) assessed via an *ex vivo* luciferase assay at 24 h after intravenous injection of LPEI (N/P 9) and succPEI (w/w 1.5) polyplexes, resp., formed at a pDNA dose of 60 µg/animal in 200 µL of HBG buffer.

(-), fold-decrease; (+) fold-increase.

Table S3. Spleen/lung and spleen/liver luciferase expression levels.

		Luciferase expression ratios *10	
		**spleen/lung**	**spleen/liver**
LPEI	CMV	0.03	8.25
	Fscn1	0.36	96.03
succPEI	CMV	0.84	11.68
	Fscn1	11.42	176.40

Luciferase expression in organs (*n*=5; mean + SD) assessed via an *ex vivo* luciferase assay at 24 h after intravenous injection of LPEI (N/P 9) and succPEI (w/w 1.5) polyplexes, resp., formed at a pDNA dose of 60 μg/animal in 200 μL of HBG buffer.

Note: Ratios represent the quotient of RLU values of spleen and liver or lung, resp., and are multiplied by factor ten for more clarity.
